# Systematic analysis of the intersection of disease mutations with protein modifications

**DOI:** 10.1186/s12920-019-0543-2

**Published:** 2019-07-25

**Authors:** Claire M. Simpson, Bin Zhang, Peter V. Hornbeck, Florian Gnad

**Affiliations:** 1 Department of Bioinformatics and Computational Biology, Cell Signaling Technology Inc, Danvers, MA USA; 2 Massachusetts Institute of Technology, Cambridge, MA USA

**Keywords:** Posttranslational modification, Cancer, Disease, PhosphoSitePlus, TCGA, dbSNP, Signal transduction

## Abstract

**Background:**

Perturbed posttranslational modification (PTM) landscapes commonly cause pathological phenotypes. The Cancer Genome Atlas (TCGA) project profiles thousands of tumors allowing the identification of spontaneous cancer-driving mutations, while Uniprot and dbSNP manage genetic disease-associated variants in the human population. PhosphoSitePlus (PSP) is the most comprehensive resource for studying experimentally observed PTM sites and the only repository with daily updates on functional annotations for many of these sites. To elucidate altered PTM landscapes on a large scale, we integrated disease-associated mutations from TCGA, Uniprot, and dbSNP with PTM sites from PhosphoSitePlus. We characterized each dataset individually, compared somatic with germline mutations, and analyzed PTM sites intersecting directly with disease variants. To assess the impact of mutations in the flanking regions of phosphosites, we developed DeltaScansite, a pipeline that compares Scansite predictions on wild type versus mutated sequences. Disease mutations are also visualized in PhosphoSitePlus.

**Results:**

Characterization of somatic variants revealed oncoprotein-like mutation profiles of U2AF1, PGM5, and several other proteins, showing alteration patterns similar to germline mutations. The union of all datasets uncovered previously unknown losses and gains of PTM events in diseases unevenly distributed across different PTM types. Focusing on phosphorylation, our DeltaScansite workflow predicted perturbed signaling networks consistent with calculations by the machine learning method MIMP.

**Conclusions:**

We discovered oncoprotein-like profiles in TCGA and mutations that presumably modify protein function by impacting PTM sites directly or by rewiring upstream regulation. The resulting datasets are enriched with functional annotations from PhosphoSitePlus and present a unique resource for potential biomarkers or disease drivers.

**Electronic supplementary material:**

The online version of this article (10.1186/s12920-019-0543-2) contains supplementary material, which is available to authorized users.

## Background

Recent breakthroughs in next-generation sequencing technologies have been accompanied by large-scale initiatives such as the TCGA network profiling large numbers of tumors [1] or the 1000 Genomes Project cataloging human genetic variations [2]. Concordantly, DNA sequencing is becoming part of routine clinical care [3], and initiatives such as the Obama Precision Medicine program [4] aim to profile patients or healthy individuals at the molecular level via sequencing or genotyping, hereby entering the era of personalized genomics and medicine.

While these advances have outpaced our ability to functionally characterize the plethora of molecular information, recent studies showed the statistical significance of perturbed signaling and altered transferase activities at a high level [5, 6]. These results are consistent with the established classification of PTM-mediated pathways as hallmarks in cancer and other diseases [7]. Consequently small molecule inhibitors targeting kinases such as HER2 [8], RAF [9], PI3K [10], or MEK [11], have been prime targets of drug development for decades, and several of them have advanced into the clinic. In addition to kinases, recent sequencing efforts revealed other significantly altered transferases, which have been subsequently pursued as targets, including histone methyltransferase EZH2 for the treatment of myelodysplastic syndrome and cutaneous T cell lymphoma [12, 13].

However, to fully understand the impact of mutations on PTMs and pathways, in particular at the substrate level, the integration of genomic with proteomic data is required [14]. Recent studies therefore thought to identify phosphorylation network-attacking mutations in cancer cell lines [15] or to determine significantly phospho-mutated proteins and pathways in tumors [16, 17]. These analyses gave important insights into perturbed signaling, but focused on the interplay between somatic cancer mutations and phosphosites.

Here we extended previous approaches by expanding the panel of cancer types for the identification of somatic driver mutations, and focused on hotspot mutations instead of taking the entire mutation load into account. In addition we analyzed disease-associated single nucleotide polymorphisms (SNPs) from the population, and investigated other PTM types such as ubiquitylation, acetylation, and methylation. We further applied Scansite [18], a widely used method for the prediction of upstream kinase regulation, to identify rewired signaling networks. Our hypotheses on specific impacted PTM sites are backed up by functional annotations in PhosphoSitePlus [19] to form a unique resource for further investigation.

## Materials and methods

### PTM and mutation data

PTM sites with experimental evidence in human samples were retrieved from PhosphoSitePlus (version August 2018). We included PTM sites determined by low throughput methods or mass spectrometry. PTM sites, which were identified by mass spectrometry based on peptides corresponding to multiple homologous proteins (defined as ‘protein groups’) were also included in the analysis. Overall, 6.5% of the PTM sites map to protein groups. Using the CGDSR R package from cBioPortal (http://www.cbioportal.org) [20, 21] missense mutations from tumors across the following TCGA cancer types were retrieved: bladder carcinoma (BLCA) [22], breast carcinoma (BRCA) [23], colorectal carcinoma (COADREAD) [24], glioblastoma (GBM) [25], head and neck squamous cell carcinoma (HNSC) [26], chromophobe renal cell carcinoma (KICH) [27], clear cell renal carcinoma (KIRC) [28], acute myeloid leukemia (LAML) [29], lung adenocarcinoma (LUAD) [30], lung squamous cell carcinoma (LUSC) [31], ovarian carcinoma (OV) [32], prostate adenocarcinoma (PRAD) [33], gastric carcinoma (STAD) [34], papillary thyroid carcinoma (THCA) [35], and endometrial carcinoma (UCEC) [36]. Annotated human missense variants were downloaded from Uniprot (https://www.uniprot.org) (humsavar.txt, release 2018_02) [37]. Of these, disease-associated germline variants that are also recorded in the dbSNP database [38] were selected for further analyses. The vast majority of variants in dbSNP (> 99.8%) have been classified as germline variants, so that we define the dbSNP dataset as the source for ‘germline’ mutations.

### Mutation analyses

Hotspot mutation scores (ΔS) were calculated for each protein as described [39]:

Where *n* is the total number of mutations, *k* is the number of different mutation types, *n*_*i*_ is the number of occurrences for mutation *i*, and *f*_*i*_ is the frequency of mutation *i* or *n*_*i*_ */n*.

$$ S=\sum \limits_{i=1}^k-{f}_i\bullet \ln \left({f}_i\right) $$

$$ {S}_0=\sum \limits_{i=1}^k-{p}_i\bullet \ln \left({p}_i\right)=\ln (k) $$

$$ \Delta S={S}_0-S $$

For the clustering and comparison of frequencies of amino acid changes each missense alteration type (from one amino acid to any of 19 others) was counted in both the somatic and germline datasets, and categorized by the unmodified wild-type amino acid or by the impacted PTM class. The resulting count matrix was normalized and used as input to create heatmaps with the R package *pheatmap* version 1.0.10 (https://cran.r-project.org/package=pheatmap). Default parameters (complete hierarchical clustering and Euclidean distance) were used for row- and column-wise clustering. Expected mutations on PTM sites were calculated by taking the product of the number of observed mutations on the unmodified amino acid and the proportion of those amino acid residues that are a PTM site in the human proteome.

### Prediction of altered upstream kinase regulation

Somatic hotspot and germline SNP mutations within 5 residues of a PTM site were compiled, and mutated flanking sequences (+/− 7 residues) were derived. Scansite 4.0 [18] was used to calculate kinase-binding scores corresponding to wild type or mutated flanking sequences at minimum stringency. DeltaScansite scores were defined as the difference between the Scansite scores for mutated and wild type flanking sequences. A second set of scores corresponding to the same wild type and mutated flanking sequences was calculated using RMIMP (version 1.2) [40]. Predictions were matched for rewiring events for which both methods provided scores.

## Results

### Integrating PTM sites with disease variants

**Fig. 1:**
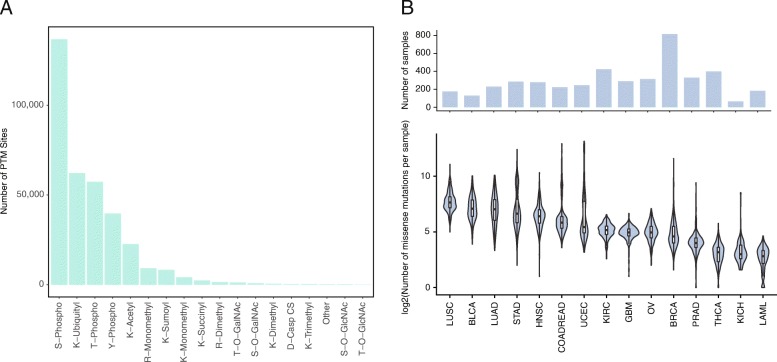
Overview of PhosphoSitePlus and TCGA datasets. **a** Total number of human PTM sites by PTM type in PhosphoSitePlus. **b** Bar plot showing total number of tumors and violin plot showing the distribution of the number of missense mutations per tumor for each TCGA cancer type

We retrieved PTM sites from PhosphoSitePlus (https://www.phosphosite.org), somatic mutations from thousands of TCGA tumors from cBioPortal, and disease-associated SNPs from Uniprot and dbSNP (Materials and Methods). Altogether, we collected 348,570 PTM sites on 18,154 human proteins. The most frequent modification sites included 234,058 phosphorylation sites, 62,216 ubiquitylation sites, 22,712 acetylation sites, and 15,872 methylation sites (Fig. 1a). The TCGA dataset contained 481,370 somatic missense mutations from 4440 tumors across 15 cancer types (Fig. 1b). Filtering the dbSNP dataset for disease-associated human variants, which result in missense alterations at the protein level, yielded a set of 18,511 non-redundant mutations on 2532 proteins linked with more than 3000 different diseases.

### Identification of somatic hotspot mutations reveals potential cancer drivers

**Fig. 2:**
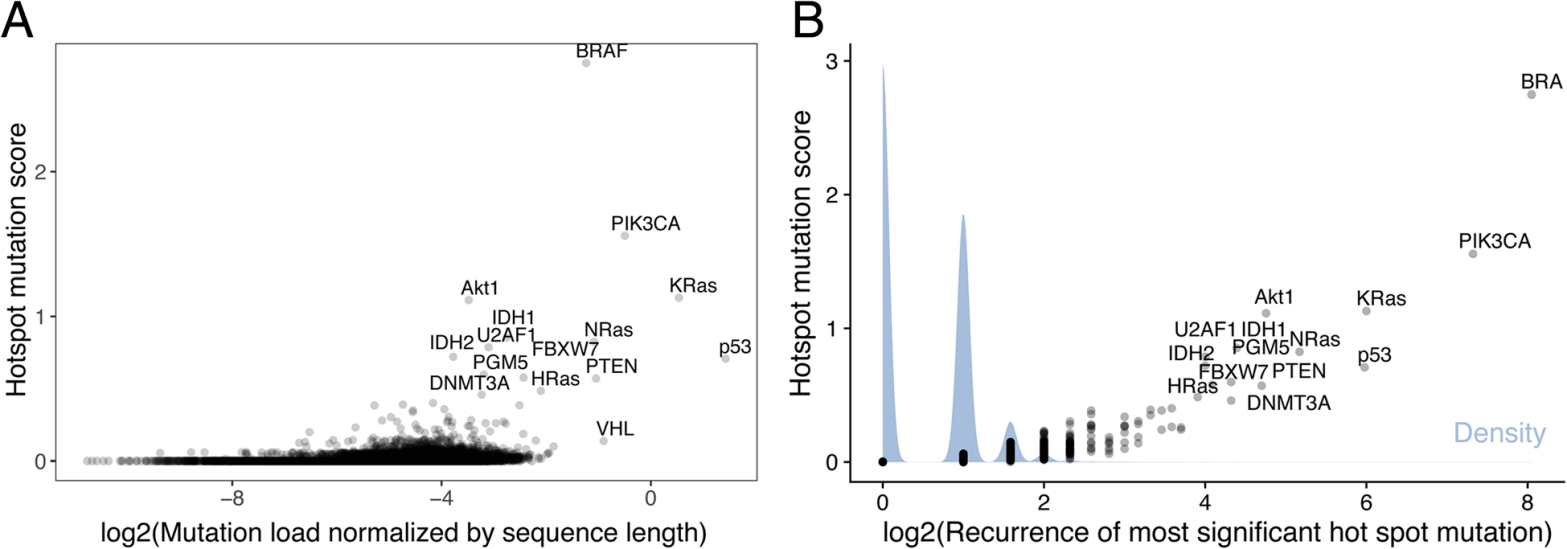
Correlation of hotspot mutation score (delta S) and the number of mutations. **a** The logarithm of the number of mutations found in a protein normalized by its sequence length plotted against its hotspot mutation score. **b** The logarithm of the number of times the most frequent alteration occurs in a protein plotted against its hotspot score. The associated density is in blue

While the set of germline variants exclusively contained disease-associated SNPs and hence did not require further filtering, only a fraction of somatic mutations in the TCGA dataset are tumorigenic. To distinguish between passenger and driver mutations, we determined recurrent mutations and calculated entropy-based ‘hotspot mutation scores’ [39] reflecting the preferred occurrence of specific point mutations in a protein. Hotspot mutations are likely cancer-driving, and present the most characteristic feature of oncoproteins [41]. As expected, known cancer proteins BRAF, PIK3CA, KRas, Akt1, IDH1, and NRas showed the highest hotspot mutation scores in the TCGA dataset (Fig. 2 and Additional file 1). However, many other proteins, whose contributions to oncogenesis are unknown or not fully understood, also revealed hotspot mutations. For example, splicing factor U2AF1 showed a recurrent mutation (S34F) in leukemia and lung adenocarcinoma resulting in the 7th highest hotspot score in our analysis. Similarly, phosphoglucomutase-like protein 5 (PGM5) had a hotspot mutation (I98V) in stomach cancer and the 10th highest score.

**Fig. 3:**
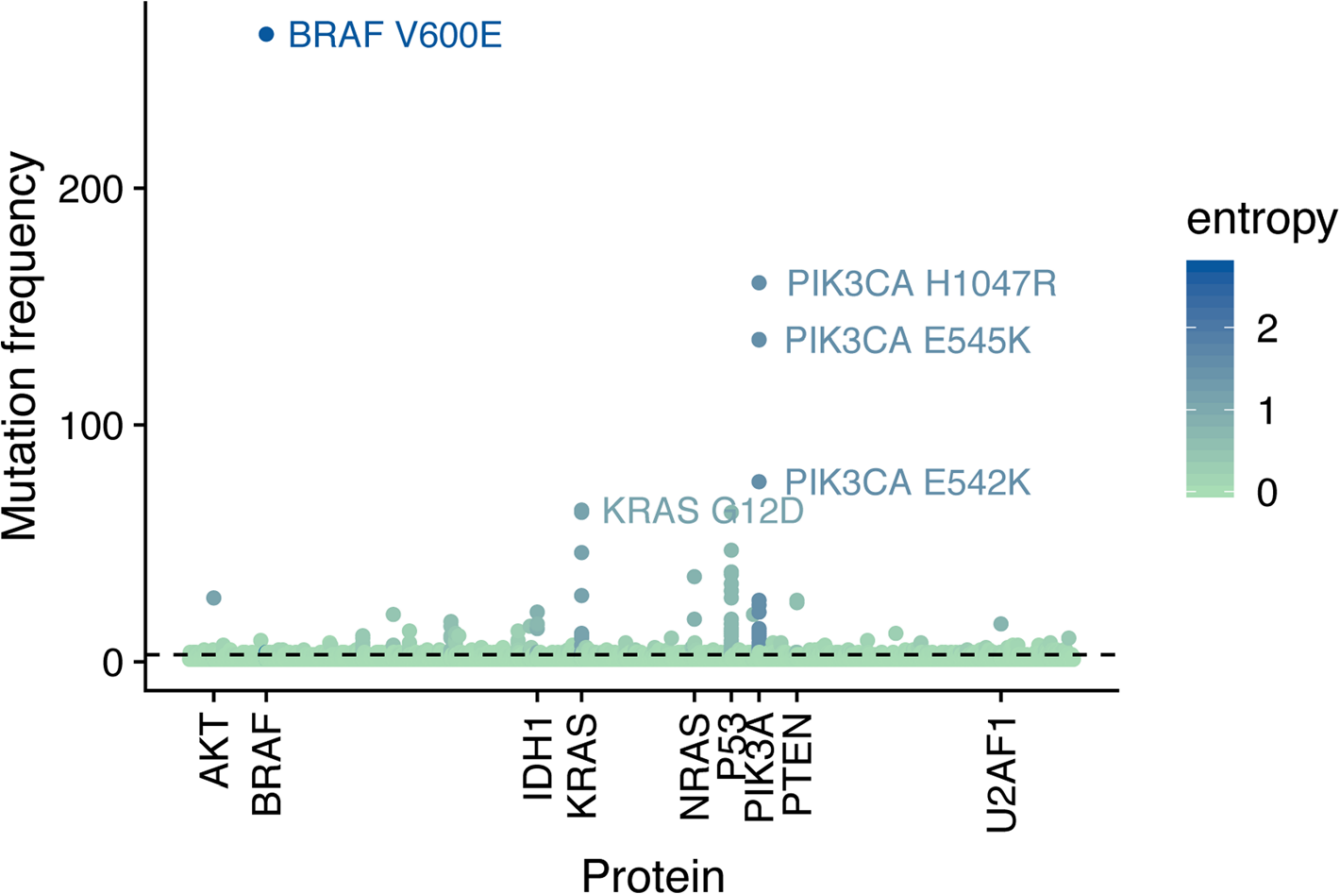
Manhattan plot of TCGA alterations. Each alteration in the TCGA dataset is plotted by its frequency. Light green indicates a low hotspot mutation score for the associated protein, while dark blue indicates a high hotspot mutation score. The dashed line indicates the 3-tumor cutoff used to define hotspot mutations

By definition, hotspot scores at the protein level correlate with the degree of recurrence of corresponding mutations. The presence of at least one recurrent mutation found in three or more tumors was sufficient for a protein to gain a hotspot score significantly higher compared to proteins with less recurrent mutations (*p* < 0.01 based on Mann-Whitney-Wilcoxon test) (Fig. 2b). Using this cutoff to enrich for oncogenic mutations yielded a set of non-redundant 1783 hotspot mutations on 1369 proteins (Fig. 3, Additional file 2). The most frequent hotspot mutations on oncoproteins included V600E in BRAF (265 tumors), H1047R (160 tumors), E545K (136 tumors), and E542K (76 tumors) in PIK3CA, G12D (64 tumors) and G12 V (63 tumors), G12C in KRas (46 tumors), and Q61R in NRas (36 tumors). Strikingly, hotspot mutations also occurred in tumor suppressors, which are known to be enriched for loss-of-function mutations almost evenly distributed along the protein sequence [39]. p53 showed the most frequent mutations including R175H (63 tumors), R273H (47 tumors), R248W (38 tumors), and R248Q (37 tumors), followed by PTEN containing hotspot mutations such as R130G (26 tumors) and R130Q (25 tumors). The unexpected presence of hotspot mutations in tumor suppressors has been investigated in previous studies [42], but most cases are not fully understood. Notably the number of tumors per cancer type varied from 65 (chromophobe renal cell carcinoma) to 817 (breast cancer), so that the ranking of hotspot mutations by frequency was biased towards cancer types with larger cohorts. We therefore included cancer type-specific scores in Additional file 1.

### Several proteins reveal high densities or frequencies of disease mutations

Prior to merging the PTM data with the disease datasets, we sought to compare the characteristics of somatic cancer mutations versus germline mutations associated with various diseases. Consistent with the approximately 200-fold wider range of diseases, the germline set contained around 10-fold (1783/18511) more mutations but only 2-fold (1369/2532) more proteins than the somatic set. This discrepancy traces back to proteins showing numerous disease-associated germline mutations (Additional file 3 A and B). Protein SCN1A showed the largest number of distinct disease variants (219 genetic variants associated with five different diseases). Taking the protein sequence into account protein PAH showed the highest density of variants (213 variants within 452 residues associated with 3 different diseases). In comparison, in the TCGA dataset Titin showed the largest total number of variants (2006 mutations across all patients), and p53 contained the highest density of variants (1058 mutations across all patients within 393 residues). Additionally, p53 contained both the largest total number and highest density of unique hotspot mutations, with 96 unique hotspot alterations on p53 found in the dataset.

### Deleted PTM sites in diseases

**Fig. 4:**
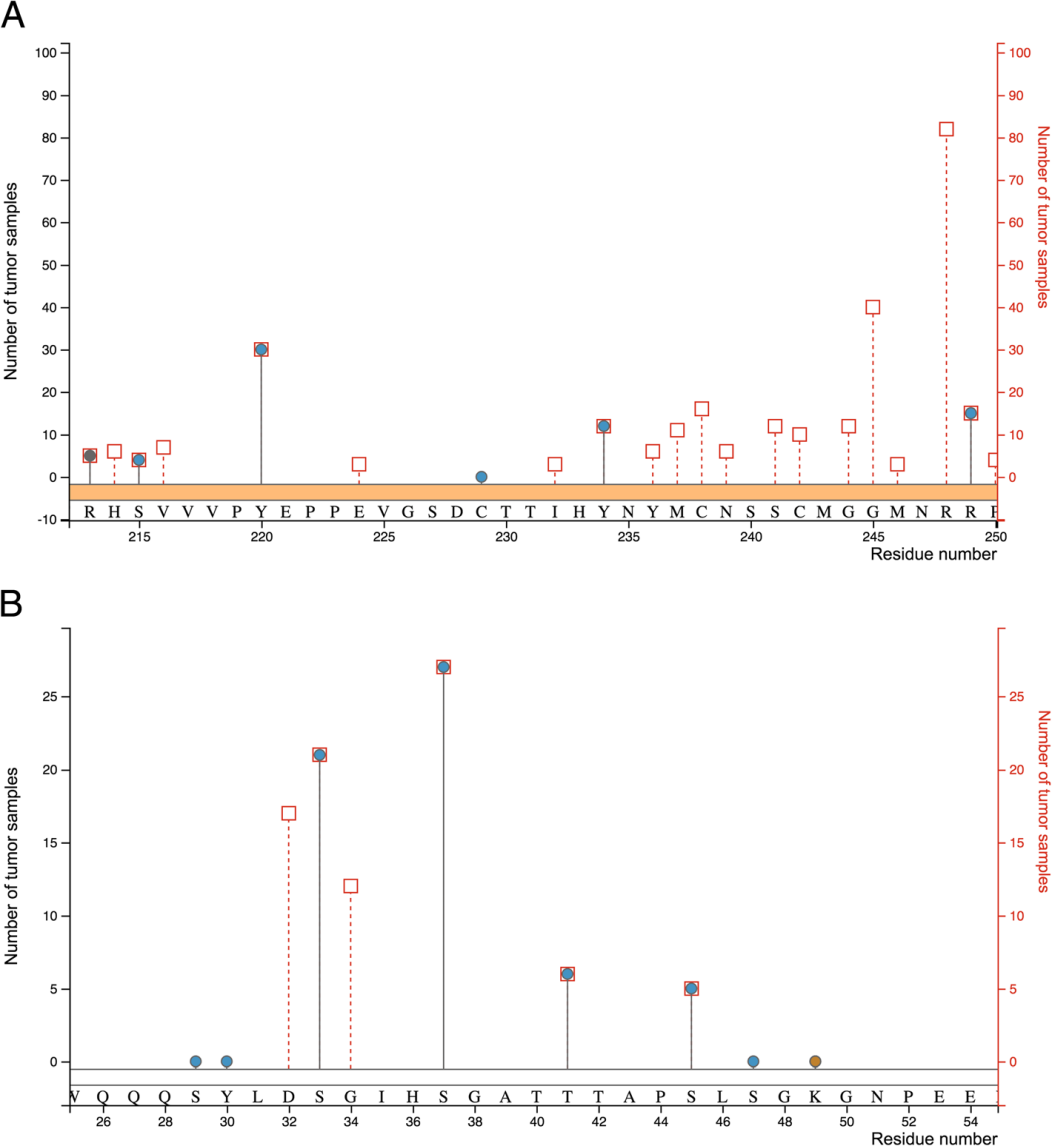
PhosphoSitePlus lollipop plots of (**a**) p53 and (**b**) CTNNB1. Circles indicate PTM sites with a height reflecting the number of references describing the site. Squares indicate hotspot mutations with a height reflecting the number of TCGA tumors containing mutations on that residue

Having filtered and characterized somatic hotspot mutations and disease-associated mutations from dbSNP, we set out to identify mutations directly affecting PTM sites. For this analysis, we postulate that previously reported PTM events would indeed be present in a tissue in the absence of mutations. First, we mapped TCGA mutations and PTM sites to the corresponding protein sequences and investigated the overlap. We found 49 somatic hotspot mutations in TCGA, destroying 45 PTM sites in 35 proteins (Additional file 4). This overlap between PTM sites and somatic mutations was significantly larger than expected (*p* < 0.01 based on a two-tailed Fisher exact test). While p53 (7 PTM sites) (Fig. 4a), CTNNB1 (4 PTM sites) (Fig. 4b), and hnRNP U (2 PTM sites) showed multiple impacted sites, all other proteins had only one lost PTM site including known cancer proteins such as EGFR, APC1, BRAF, or RAF1. A total of ten affected PTM sites have been associated with specific biological processes or molecular functions. For example, phosphorylation of proto-oncoprotein beta-catenin on S33, S37, and T41 by GSK-3 beta is known to target beta-catenin towards degradation, and CK1-mediated phosphorylation of S45 functions as a gatekeeper for this process [43]. Mutations of these sites primarily occur in endometrial but also other cancer types including lung cancer. The destruction of these regulating PTM sites presumably prohibits the degradation process leading to continuous and oncogenic activity of beta-catenin. While the loss of these PTM sites on beta-catenin are consistent with an oncogenic model, other cases are more complex. For example, PRMT1-mediated methylation of EGFR on R222 has been shown to enhance binding to EGF and subsequent receptor dimerization and signaling activation [44]. However, the loss of this PTM site, found in 1.4% of glioblastoma samples, would be consistent with a tumor-suppressing role of the mutation. Overall, most cases, such as the mutation on growth factor receptor-bound protein 10 (T422 M), found in three tumors, have been detected by mass spectrometry without functional characterization, forming a candidate set for further characterization.

**Fig. 5:**
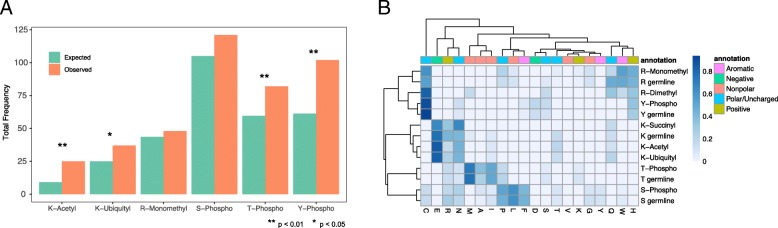
Comparison of alterations found on PTM sites and all alterations. **a** Barplot comparing the number of observed (orange) to expected (green) mutated PTM sites in the SNP dataset. **b** Heatmap of the frequencies of alteration types within the germline datasets on PTM sites and their unmodified counterparts, normalized by row. Mutation residues are annotated by residue type

We also examined the set of disease-associated germline SNPs, and identified 420 genetic variants overlapping with 402 PTM sites on 276 proteins (Additional file 5). A total of 73 proteins showed two or more overlaps between PTM sites and germline mutations. Lamin A/C (20 overlaps), CTNNB1 (10 overlaps), and p53 (9 overlaps) showed the largest number of intersects. The observed number of SNP mutations on K-acetylation, T-phosphorylation and Y-phosphorylation sites was significantly greater than expected (*p* < 0.01 based on a two-tailed Fisher exact test) (Fig. 5a). A total of 48 destructed PTM sites have been functionally characterized by previous studies (Additional file 5). Among these, the best-described PTM site is S32 on NF-kappa-B inhibitor alpha (IkB-alpha). Overall, 127 references have described the functional impact of phosphorylation of IkB-alpha at S32 leading to proteasome-mediated degradation and consequent activation of NF-kappa-B/Rel transcription factors via translocation from the cytosol to the nucleus [45]. Mutation on this residue has been shown to be associated with autosomal dominant anhidrotic ectodermal dysplasia and T cell immunodeficiency [46]. As observed for the overlap with somatic mutations, most dbSNP-overlapping PTM sites have been experimentally validated without functional characterization including PTM sites on cancer proteins. For example, phosphorylation of RAF1 on T310 has been validated in six high-throughput experiments. While the function of this PTM site is unknown, variation on the residue (T301A) is associated with childhood-onset dilated cardiomyopathy [47].

### Mimicked phosphorylation sites in diseases

While the destruction of PTM sites in diseases might imply the loss of tumor-suppressing functions in the cell, constant activation of PTM sites points to the promotion of oncogenic processes. Focusing on phosphosites, we scanned the data for residues mutated to the negatively charged amino acids aspartic (D) and glutamic acid (E). This was based on the idea that diseases might utilize the same trick that scientists use in experiments to mimic constantly active phosphosites [48]. While none of the overlapping somatic hotspot mutations contained mimicking alterations, eleven SNPs intersected with phosphosites in their wild type form and mimicked them in diseases (Additional file 6). Interestingly, all of these were phosphorylated tyrosines mutated to aspartic acid. Strikingly, phosphorylation of non-receptor-type protein tyrosine phosphatase SHP-2 on Y62 has been detected in 2115 mass spectrometry experiments, but not functionally characterized to the best of our knowledge. The overlapping mutation (Y62D), however, has been associated with Noonan Syndrome [49].

In addition to the identification of phosphosites that are phosphorylated in the wild type state and mimicked in mutated form, we identified 31 somatic hotspot and 1230 germline mutations that mutated to aspartic and glutamic acid – irrespective of whether they present PTM sites in their wild type origin. Besides well-known cases such as BRAF (V600E), KRas (G12D, G13D) or PIK3CA (G118D), we derived previously uncharacterized hotspot mutations such as SF3B1 (K700E) found in eight breast tumors and one leukemia sample.

We also looked into the distributions of each kind of missense mutation on PTM sites compared to unmodified residues. Clustering of these frequencies showed that mutation patterns for PTM sites were similar to their unmodified counterparts in the TCGA (Additional file 7) and the dbSNP sets (Fig. 5b).

### Mutations triggering rewiring of signaling networks

While mutations on central PTM sites trigger their loss or in some cases even mimic their active states, mutations in their flanking regions could rewire regulation by changing the substrate sequence motif. Scansite uses scoring matrices derived from peptide library experiments to identify short protein sequence motifs [18]. For the first time we applied Scansite on wild type and mutated flanking regions of PTM sites to derive ‘Delta-Scansite’ score as a measure of rewiring (Materials and Methods). We also compared the results with the machine learning method MIMP [40].

**Fig. 6:**
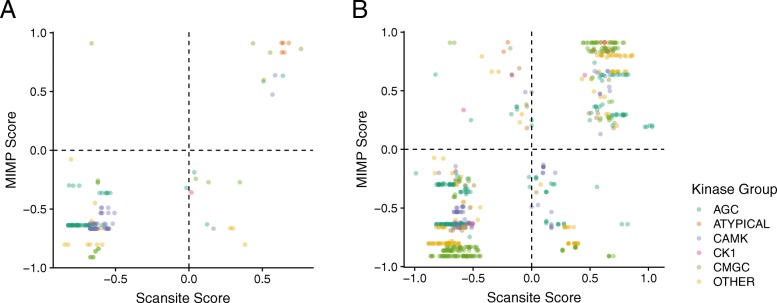
Comparison of Delta-ScanSite and MIMP scoring for (**a**) TCGA hotspot mutations and (**b**) disease SNPs in flanking regions of PTM sites. The difference between predicted mutated and wild type kinase binding scores for ScanSite is plotted against the difference in scores calculated by MIMP. Dots are colored according to the group of the kinase

In the TCGA dataset, Delta-Scansite and MIMP calculated 149 matching rewiring scores for 87 mutations on 83 flanking regions across 73 proteins. Among these paired scores, 138 (93%) agreed in sign (Fig. 6a and Additional file 8 A). For example, both Delta-Scansite and MIMP predicted a loss phosphorylation of T284 on p53 by Aurora B due to the alteration R282W found in 30 tumors across multiple cancer types. Aurora B-mediated phosphorylation on T284 has been shown to compromise p53 transcriptional activity [50].

Similarly, both methods predicted a loss phosphorylation of T254 on MTF1 by Akt1. Dephosphorylation of T254 by phosphatase PP2A PR110 has been shown to regulate MTF1 activity [51]. To our knowledge the corresponding kinase, however, has not been reported. Furthermore, in addition to mutations leading to direct loss of regulating PTM sites of beta-catenin as described above, both approaches predicted reduced phosphorylation on S33 by GSK, triggered by three hotspot mutations on a flanking residue that is itself a phosphosite (S37C, S37F, and S37A). These mutations were found in primarily endometrial cancer tumors.

In the SNP dataset, 590 scores were calculated for 318 mutations on 283 sites across 188 proteins (Additional file 8 B). In total, 494 (84%) of these scores agreed in sign (Fig. 6b). For example, the S112 phosphosite on PPAR-gamma plays a role in cell differentiation, growth, and transcription as described by multiple studies curated in PhosphoSitePlus, and the flanking SNP on P113Q has been associated with obesity [52]. Both our approach and MIMP predicted a loss of MAPK and CDK binding to S112, but a gain of ATM Kinase binding, indicating a possible rewiring event.

While most predictions were concordant between DeltaScansite and MIMP, we also observed contradictory predictions. For example, MIMP predicted that mutations of arginine to histidine on the − 3 position relative to AKT1 substrate sites induce loss of phosphorylation by AKT1. In contrast DeltaScansite predicted gain of phosphorylation. The canonical sequence motif for AKT substrates requires an arginine on the − 3 position. However, AKT has been indeed reported to potentially phosphorylate SP1 at T679 despite histidine on the − 3 position [53]. Thus it is difficult to determine which prediction method is correct in such cases.

Altogether we found that DeltaScansite and MIMP predictions are consistent. The rewiring events suggested by these methods provide a unique resource for studying perturbed signaling in diseases.

## Concluding remarks and future plans

The convergence of our knowledge about missense mutations and PTMs has opened up a new approach for analyzing and understanding the interplay between disease mutations and cellular signaling networks. This interplay provides a unique framework for investigating pathogenesis initiated by missense mutations, and conversely for understanding the cellular processes and signaling networks influenced by the posttranslational status of a modification site.

We have analyzed the union of somatic mutations in 15 different cancer types, disease-associated germline mutations, and PTMs. To our knowledge this is the first study to include acetylation, methylation, ubiquitylation, and other non-phosphorylation PTM sites in the analysis of the intersection with mutations. In fact we found more than one hundred non-phosphorylation PTM sites overlapping with disease mutations. In addition to disease-associated germline variants we included somatic cancer mutations. Distinguishing between passenger and driver mutations based on recurrence revealed a number of somatic hotspot mutations previously not linked with tumorigenesis. While mutations on central PTM sites presumably result in the destruction or even mimicking of their active states, mutations in the flanking sequence motif could rewire regulation as predicted by Scansite and MIMP.

We discovered mutations that may impact posttranslational signaling, modifying protein function and network dynamics. Our datasets serve as unique resources for potential biomarkers or disease drivers. The concordance between DeltaScansite and MIMP makes clear that altered upstream regulation can be estimated *in-silico* and extended for any PTM type in the future.

## Additional files

Additional file 1: The hotspot score (delta S) and number of somatic alterations in each protein. (XLSX 2740 kb)

Additional file 2: Hotspot mutations found in the TCGA dataset. (XLSX 152 kb)

Additional file 3: Comparison of TCGA and SNP alterations. (**A**) Density plot of the distribution of the number of SNPs in each protein. (**B**) Density plot of the distribution of the number of somatic hotspot alterations in each. (PDF 138 kb)

Additional file 4: Hotspot mutations on PTM sites found in the TCGA dataset. (XLSX 55 kb)

Additional file 5: Disease SNPs overlapping with PTM sites. (XLSX 85 kb)

Additional file 6: Phosphosite-mimicking (**A**) hotspot mutations and (**B**) disease SNPs. (C) Disease SNPs on phosphosites mimicking constitutive activation. (XLSX 105 kb)

Additional file 7: Heatmap of the frequencies of alteration types within the somatic datasets on PTM sites and their unmodified counterparts, normalized by row. Mutation residues are annotated by residue type. (PDF 8 kb)

Additional file 8: Delta-Scansite predictions for (**A**) somatic and (**B**) germline alterations in flanking regions of PTM sites. (XLSX 105 kb)

## Data Availability

PhosphoSitePlus (https://www.phosphosite.org) provides information about PTM sites and mutations for each protein.

## Abbreviations

PSP: PhosphoSitePlus
PTM: Posttranslational modification
SNP: Single nucleotide polymorphism
TCGA: The Cancer Genome Atlas
