# Supplementary figures and images for: Systematic analysis of the intersection of disease mutations with protein modifications
Source: BMC Med Genomics. 2019 Jul 25;12(Suppl 6):109. doi: 10.1186/s12920-019-0543-2 (PMC6657027; doi:10.1186/s12920-019-0543-2)

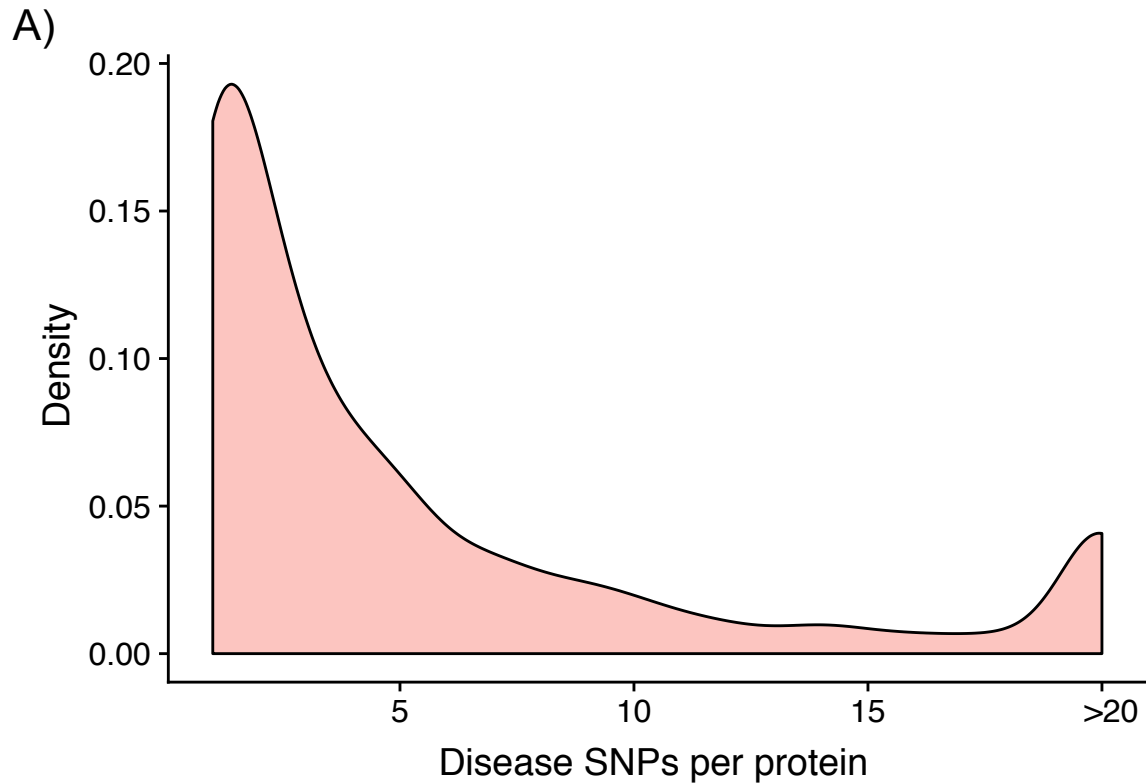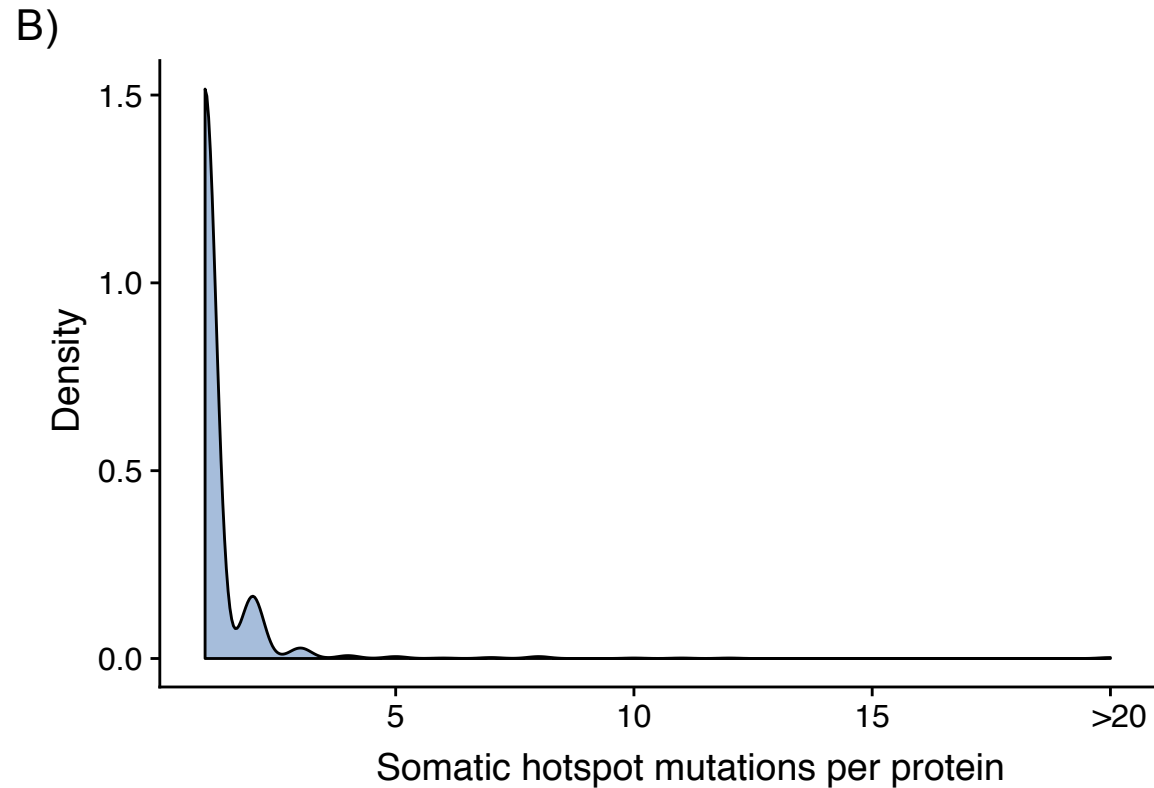

Supplement: Supplementary file 3 — Comparison of TCGA and SNP alterations. (A) Density plot of the distribution of the number of SNPs in each protein. (B) Density plot of the distribution of the number of somatic hotspot alterations in each. (PDF 138 kb) [file 12920_2019_543_MOESM3_ESM.pdf]

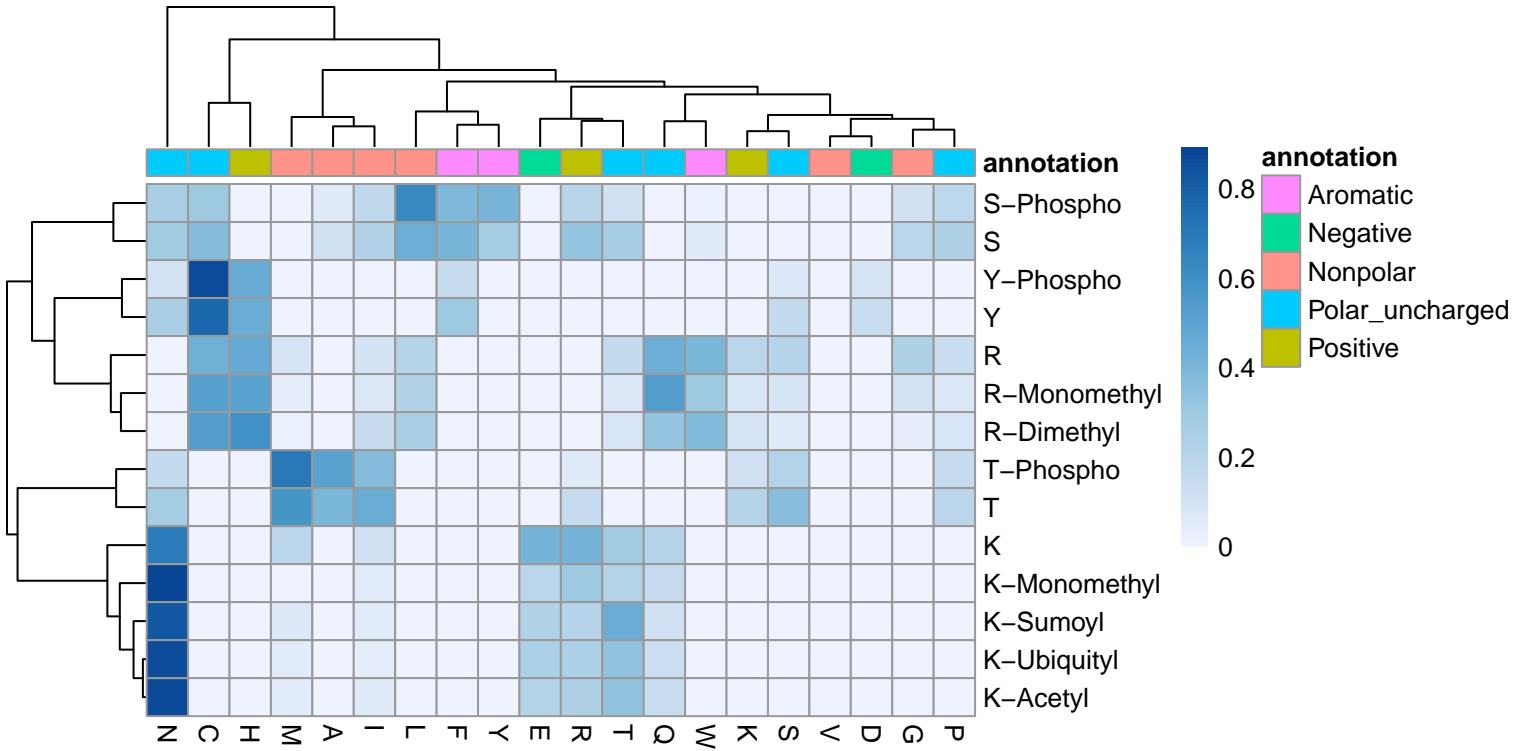

Supplement: Supplementary file 7 — Heatmap of the frequencies of alteration types within the somatic datasets on PTM sites and their unmodified counterparts, normalized by row. Mutation residues are annotated by residue type. (PDF 8 kb) [file 12920_2019_543_MOESM7_ESM.pdf]
